# Clinical and Radiologic Outcomes of Arthroscopic Rotator Cuff Repair in Medial Bursal-Side Fosbury Flop Tears Compared With Tendinous Avulsion Lesions

**DOI:** 10.1016/j.asmr.2023.100879

**Published:** 2024-02-09

**Authors:** Sean W.L. Ho, Tiago Martinho, Arash Amiri, Jeanni Zbinden, Xue Ling Chong, Hugo Bothorel, Philippe Collin, Alexandre Lädermann

**Affiliations:** a Department of Orthopedic Surgery, Tan Tock Seng Hospital, Singapore; b Division of Orthopaedics and Trauma Surgery, Hôpital de La Tour, Meyrin, Switzerland; c Royal Square Medical Centre, Singapore; d Research Department, Hôpital de La Tour, Meyrin, Switzerland; e American Hospital of Paris, Neuilly-sur-Seine, France; f Faculty of Medicine, University of Geneva, Geneva, Switzerland; g Division of Orthopaedics and Trauma Surgery, Department of Surgery, Geneva University Hospitals, Geneva, Switzerland

## Abstract

**Purpose:**

To determine the clinical and radiologic outcomes after surgical repair of medial bursal-side Fosbury flop rotator cuff tears compared with traditional avulsion of tendinous attachments lesions.

**Methods:**

A retrospective cohort study was performed. All patients who had undergone arthroscopic posterosuperior repair were recruited. Patients with previous shoulder rotator cuff surgery were excluded. Recruited patients were divided into 2 groups: one presenting Fosbury flop tears and the other presenting with standard avulsion lesions. Preoperative demographics such as age, gender, and arm dominance were recorded. Range of motion (ROM), visual analog scale (VAS) for pain and satisfaction, Constant score, Single Alpha-Numeric Evaluation score, and American Shoulder and Elbow Surgeons score were evaluated at 3 points in time: preoperatively, and at 6 months and minimum 1-year postoperatively. The healing of repaired cuffs was evaluated by ultrasound at 6 months.

**Results:**

Two hundred thirty-six patients were recruited, with 27 (11.4%) Fosbury flop tears and 209 (88.6%) tendon avulsions. Although there was no significant difference in gender or arm dominance between the groups, Fosbury flop tears had significantly older patients (*P* < .05) with a mean age 61.6 years (standard deviation 9.0), compared with tendon avulsions with a mean age of 56.1 years (standard deviation 9.1). There was no significant difference in tendon retraction between the groups. Both groups demonstrated significant improvement in ROM, visual analog scale, American Shoulder and Elbow Surgeons, Single Alpha-Numeric Evaluation, and Constant score postoperatively at 6 months and minimum 1 year. The groups demonstrated no significant difference in the ROM and clinical scores. There was a nonsignificant difference in re-tear rate of 7.4% (2/27) in Fosbury flop tears compared with 2.8% (6/209) in tendon avulsions (*P* = .361).

**Conclusions:**

Arthroscopic rotator cuff repair of medial bursal side Fosbury Flop rotator cuff tears results in favorable clinical and radiologic outcomes at 4 years after surgery. These outcomes are comparable with surgically repaired avulsion lesions, with an acceptable retear rate after arthroscopic repair.

**Level of Evidence:**

Level III, retrospective comparative prognostic trial.

Full-thickness superior rotator cuff lesions can be classified as avulsion of tendinous attachments, midsubstance tear, Fosbury flop tear, or postoperative bony adhesions.^1^ A Fosbury flop tears lesion is thus a variant of a full-thickness tear in the posterosuperior region of the rotator cuff (Fig 1).^2^ It is defined as a tear of the rotator cuff that has flipped onto itself, resulting in the flipped portion of the cuff tendon healing onto itself. This morphology is uncommon, with a reported incidence of 2.6% to 7.9%.^2^, ^3^, ^4^ Specific magnetic resonance imaging (MRI) features have been described for Fosbury flop tears (Fig 2).^4^ These include a thickened tendon of more than 9 mm (which occurs as the tendon heals upon itself and increases the apparent thickness of the tendon), visualization of a tendon stump with anteromedial orientation, and fluid accumulation in the superomedial part of the subacromial bursa.^4^ Preoperative imaging can allow the surgeon to assess the cuff tear for a possible Fosbury flop tears, for failure to acknowledge this lesion intraoperatively may result in inadvertent debridement of the viable flipped rotator cuff tendon. This, in turn, can lead to an iatrogenically shortened rotator cuff tendon, which may not be reducible to the anatomical footprint or may only be reduced under excessive tension.

**Fig 1:**
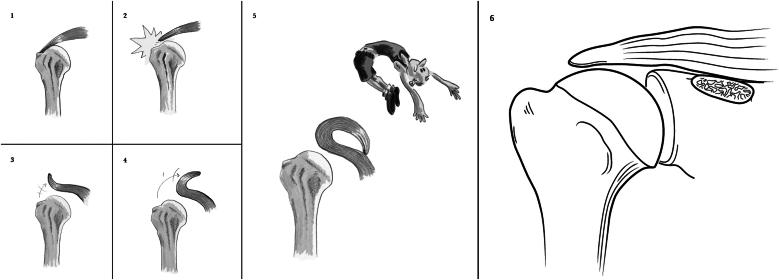
Images 1-5 represent a coronal view of a right shoulder with the development of a Fosbury flop tear. Image 6 illustrates an avulsion of tendinous attachments.

**Fig 2:**
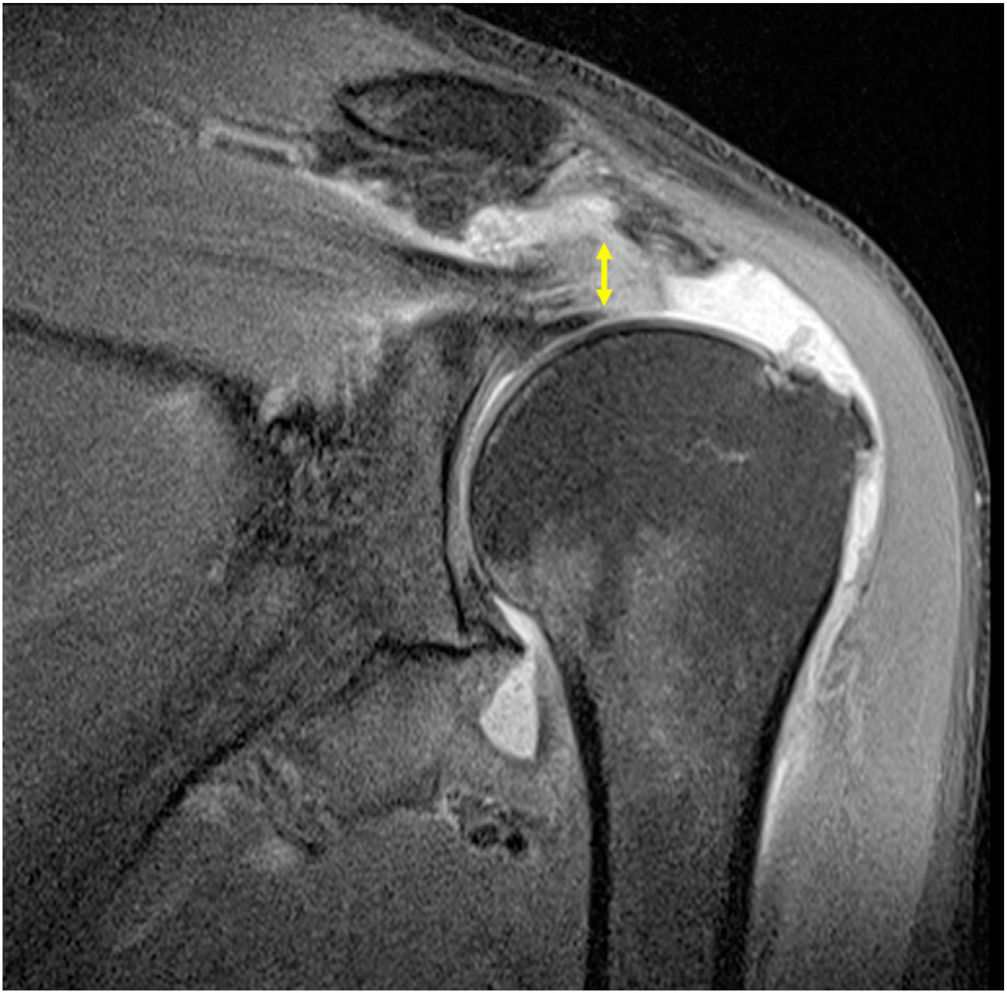
A coronal proton-density magnetic resonance imaging with fat saturation of a left shoulder shows a retracted supraspinatus tear with an abnormally thickened tendon stump (10 mm, yellow arrow), consistent with a possible Fosbury flop tear.

Awareness of this tear variant can help surgeons to apply the appropriate surgical techniques to release the flipped fragment and secure this viable tendinous tissue to the greater tuberosity footprint. There has been increased awareness of the Fosbury flop tears within the published literature.^2^, ^3^, ^4^, ^5^ However, there remains a paucity of literature assessing the clinical outcomes of patients with Fosbury flop tears who have undergone arthroscopic rotator cuff repair. As such, the clinical implications of Fosbury flop tears and their distinction from avulsion of tendinous attachments are uncertain.

The purpose of this study was to determine the clinical and radiologic outcomes after surgical repair of medial bursal-side Fosbury Flop rotator cuff tears compared with traditional avulsion of tendinous attachments lesions. We hypothesized that arthroscopic repair of medial bursal side Fosbury Flop rotator cuff tears would yield poorer results compared with repairs of tendinous avulsion lesions.

## Methods

### Patient Selection

Between January 2016 and November 2021, all patients who had undergone superior rotator cuff repair performed by the senior author (A.L.) were considered potentially eligible for inclusion in this retrospective study. The inclusion criteria included patients aged 18 years to 80 years, Collin type D rotator cuff tears,^6^ and rotator cuff muscle fatty infiltration limited to Goutallier grade 0 to 2. Patients with the following conditions were excluded: revision rotator cuff surgery,^7^ shoulder pathologies such as previous fractures or progressive glenohumeral osteoarthritis, rotator cuff muscle fatty infiltration Goutallier grade 3 or 4,^8^ preoperative infection, psychiatric problems that precluded informed consent or inability to read or write, partial repairs,^9^ or incomplete documentation. The eligible patients were divided into 2 groups: Fosbury flop tears (confirmed intraoperatively) and tendon avulsion. Ethics approval was obtained by the local ethics committee (AMG 12-26). All patients gave informed written consent.

### Surgical Technique for Fosbury Flop Tears

**Fig 3:**
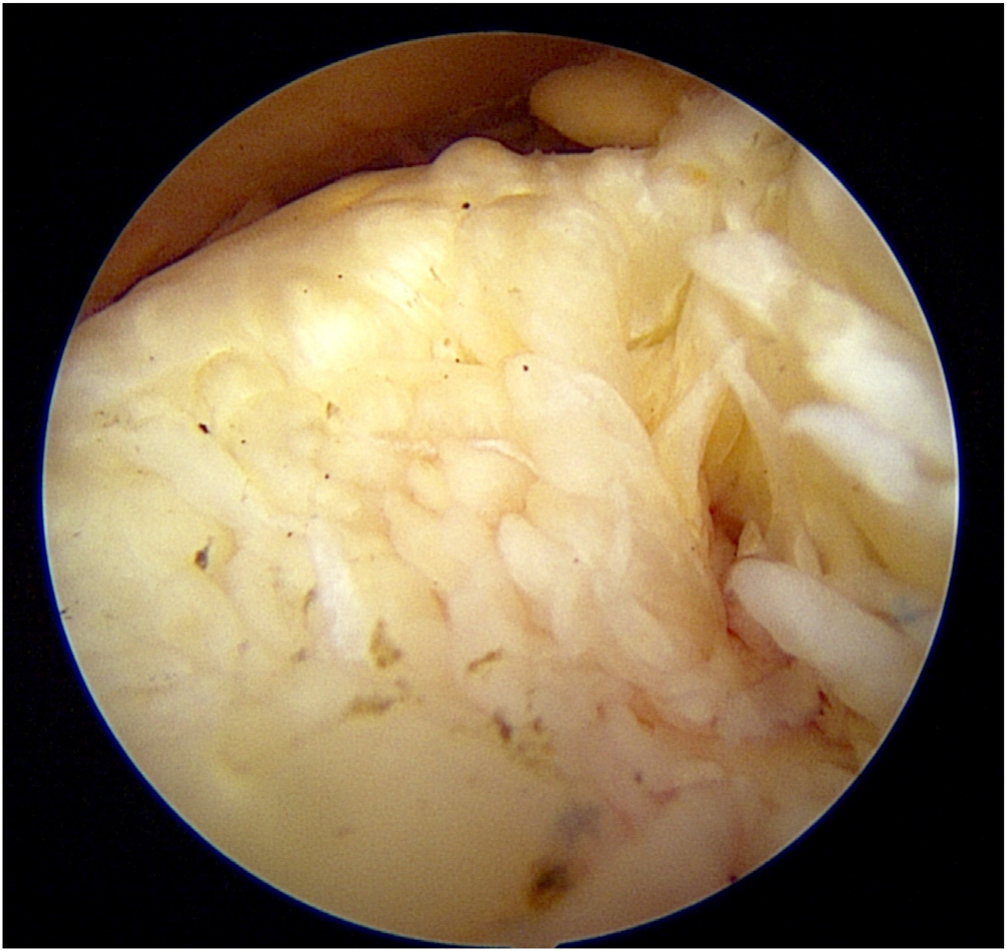
Subacromial arthroscopy of a right shoulder demonstrates the “sea-anemone” appearance of the flipped segment of a Fosbury flop tear.

All patients underwent surgery by the senior author (A.L.). Patients were positioned in the beach-chair position, with an interscalene nerve block as well as general anesthesia. A standard posterior viewing portal was used for glenohumeral joint inspection, and the biceps tendon was addressed according to the presence of biceps pathology. Tenodesis or tenotomy, where indicated, was left to the discretion of the surgeon. A view of the Fosbury flop tears, with its typical “sea-anemone” appearance, was obtained from the lateral portal. A probe was then used to confirm that the tendon had flipped upon itself (Fig 3). Once a Fosbury flop tears was identified, the flipped portion of the tendon was carefully released using radiofrequency ablation. The rotator cuff tear was then debrided, reduced, and repaired using either a single-row repair or an independent double-row technique with suture anchors.^10^ The number of anchors and surgical technique repair were determined by the size of the tear present. Adjuvant acromioplasty was performed in patients who had radiographic signs of dynamic impingement or to improve visualization during the procedure.^11^, ^12^, ^13^ Resection of the distal part of the clavicle was performed on patients who presented clinically painful acromioclavicular joints upon palpation.

A similar surgical technique was employed for the tendon avulsion. The rotator cuff tear was identified and debrided. A tension-free repair was then performed with either a single-row repair or an independent double-row technique.^10^

### Postoperative Rehabilitation

All patients received an “à la carte” postoperative rehabilitation protocol.^14^ No sling was proposed for patients who did not undergo repair under tension.^15^ Patients were allowed mobilization as tolerated with the elbow at the side. When the quality of the tendon was suboptimal, immobilization by sling was proposed for 4 to 6 weeks.^16^ A range of motion (ROM) program was then commenced at 6 weeks’ postoperatively for all types of postoperative protocols.^17^

### Clinical and Radiologic Outcome Measurements

Preoperative demographics such as age, gender, and arm dominance were recorded. At the preoperative and postoperative (6 months and minimum 1 year) examinations, the ROM, visual analog scale (VAS) for pain and satisfaction, Constant,^18^ single alpha-numeric evaluation (SANE),^19^ and American Shoulder and Elbow Surgeons (ASES)^20^ shoulder scores were evaluated. A minimum of 1 year of follow-up was chosen as such period of time is needed for patients to recover normal arm usage.^21^ ROM of the shoulder was assessed by the fellowship-trained senior surgeon (A.L.) using a goniometer. Anterior forward flexion, external rotation, and internal rotation were measured. External rotation was measured with the arm by the side of the body and internal rotation was assessed at the highest vertebral spinous process attained by the extended thumb of the patient. The level of vertebral spinous process was assessed via manual counting from the C7 vertebra. The other scores were selected for their ease of administration in addition to their well-validated assessment of shoulder function.^22^^,^^23^

Intraoperative rotator cuff tear pattern was classified according to the Collin classification.^6^ The latter divides the rotator cuff into 5 components: supraspinatus, superior subscapularis, inferior subscapularis, infraspinatus, and teres minor. The tear pattern is then classified into 5 types, depending on the location of the tear: type A (supraspinatus and superior subscapularis), type B (supraspinatus and entire subscapularis), type C (supraspinatus, superior subscapularis, and infraspinatus), type D (supraspinatus and infraspinatus), and type E (supraspinatus, infraspinatus and teres minor). Preoperative imaging by magnetic resonance imaging (MRI) also was assessed for fatty infiltration, staged according to the Goutallier classification^8^ and rotator cuff retraction, graded by the Patte classification.^24^

Healing of the repaired cuff was evaluated by ultrasound according to the Sugaya classification.^25^^,^^26^ All ultrasounds were performed at 6 months postsurgery^27^^,^^28^ by the senior surgeon (A.L.) following a published a systematic protocol.^26^ Ultrasound imaging was considered the imagery of choice for its easy accessibility and its demonstrated accuracy in the determination of postoperative rotator cuff integrity.^29^

### Statistical Analysis

A 1:3 propensity matching was performed to control for rotator cuff retraction, patient age, last follow-up duration, suture technique, biceps tendon procedure, and sling wearing after surgery. For baseline characteristics, variables were reported as proportions or mean ± standard deviation, median, and range. Shapiro–Wilk tests were used to assess the normality of distributions. For non-Gaussian continuous data, differences between groups were evaluated using Wilcoxon rank-sum tests (Mann–Whitney *U* test). For Gaussian continuous data, differences between groups were evaluated using unpaired Student *t*-test. For categorical data, differences between groups were evaluated using the χ^2^ or Fisher exact test where appropriate. Pre- to postoperative differences were evaluated using the Wilcoxon signed-rank test for non-Gaussian continuous data and using the paired Student *t*-test for Gaussian continuous data. The clinical relevance for the ASES score (principal outcome) was assessed using several thresholds published by Cvetanovich et al.^30^ minimal clinically important difference (MCID, 11.1 points), substantial clinical benefit (17.5 points), and patient acceptable symptom state (86.7 points). Statistical analyses were performed using R version 3.6.2 (R Foundation for Statistical Computing, Vienna, Austria). *P* values < .05 were considered statistically significant.

Based on a previous study performed by the same senior surgeon (A.L.), the postoperative ASES score 2 years after rotator cuff repair was reported to be 89 ± 16.^14^ A sample size calculation for the present study was performed a priori to detect a clinically MCID in ASES score (11.1 points).^30^ With a statistical power of 80% and an alpha level of 5%, 23 patients in the Fosbury flop group and 69 patients in the avulsion group were needed.

## Results

Among 813 patients, 236 had a posterosuperior rotator cuff lesion including 27 (11.4%) Fosbury flop tears and 209 (88.6%) tendon avulsion. After matching, the 2 groups comprised respectively 23 and 69 patients with available data at final follow-up. The mean follow-up was 4.4 ± 2.0 years (median, 5.0; range 1-7) years in Fosbury flop tears and 4.2 ± 1.9 years (median,4; range: 1-7) in avulsion tears (*P* = .581). No significant difference could be observed between the 2 groups except for hypercholesterolemia (*P* = .015), which was more present in the Fosbury flop group (41%) than in the avulsion group (14%) (Table 1), and preoperative internal rotation which was significantly lower in the Fosbury flop group (6 ± 4 vs 8 ± 4 *P* = .032) (Table 2).

**Table 1:** Patient Characteristics

	Avulsion Group (n = 69)		Fosbury Flop Group (n = 23)		*P* Value
	Mean ± SD or N (%)	(95% CI)	Mean ± SD or N (%)	(95% CI)	
Age, y	61.4 ± 8.6	(59.4-63.4)	63.3 ± 7.8	(60.1-66.4)	.372
BMI	25.2 ± 3.6	(24.4-26.1)	25.8 ± 3.7	(24.3-27.3)	.721
Weight, kg	76.4 ± 13.4	(73.3-79.6)	72.7 ± 12.1	(67.8-77.7)	
Height, cm	173.8 ± 8.7	(171.8-175.9)	167.9 ± 8.1	(164.6-171.2)	
Symptoms onset before surgery, mo	14.7 ± 21.1	(9.7-19.7)	16.3 ± 19.9	(8.2-24.4)	.603
Male sex	50 (72.5%)		13 (56.5%)		.197
High blood pressure∗	13 (22.8%)		8 (36.4%)		.261
Hypercholesterolemia∗	8 (14.0%)		9 (40.9%)		**.015**
Diabetes∗	1 (1.8%)		2 (9.1%)		.186
Smokers∗	15 (26.3%)		9 (40.9%)		.274
Workers’ compensation	9 (13.0%)		5 (21.7%)		.328
Traumatic origin	29 (42.0%)		7 (30.4%)		.460
Dominant arm	46 (66.7%)		16 (69.6%)		1.000
Patte classification					.287
1	8 (11.6%)		2 (8.7%)		
2	53 (76.8%)		15 (65.2%)		
3	8 (11.6%)		6 (26.1%)		
Clavicle resection	13 (18.8%)		4 (17.4%)		1.000
Adjuvant procedure					.151
None (LHB ruptured)	3 (4.3%)		4 (17.4%)		
LHB tenodesis	35 (50.7%)		10 (43.5%)		
LHB tenotomy	31 (44.9%)		9 (39.1%)		
Suture technique					.474
Single row	32 (46.4%)		13 (56.5%)		
Double row	37 (53.6%)		10 (43.5%)		
Postoperative sling wearing	69 (100.0%)		23 (100.0%)		1.000

Bold value indicates statistical significance.

BMI, body mass index; CI, confidence interval; FFT, Fosbury flop tears; LHB, long head of the biceps tendon; SD, standard deviation.

∗ Patients with available comorbidity information: n = 57 (non-FFT) and n = 22 (FFT).

**Table 2:** Clinical Outcomes

	Avulsion Group (n = 69)		Fosbury Flop Group (n = 23)		*P* Value
	Mean ± SD or N (%)	(95% CI)	Mean ± SD or N (%)	(95% CI)	
Forward flexion, °					
Baseline/preoperative	138 ± 40	(129-148)	130 ± 45	(112-148)	.251
6 mo	151 ± 22	(145-156)	151 ± 17	(144-158)	.846
Last follow-up	163 ± 20	(158-168)	167 ± 15	(160-173)	.476
External rotation (ER), °					
Baseline/preoperative	41 ± 21	(36-46)	35 ± 18	(28-43)	.185
6 mo	42 ± 19	(38-47)	39 ± 15	(33-45)	.561
Last follow-up	59 ± 24	(53-65)	65 ± 24	(55-75)	.283
Internal rotation (IR. score)					
Baseline/preoperative	8 ± 4	(7-9)	6 ± 4	(4-8)	**.032**
6 mo	8 ± 4	(7-9)	9 ± 4	(7-10)	.689
Last follow-up	10 ± 3	(9-11)	9 ± 4	(8-11)	.235
Pain on VAS					
Baseline/preoperative	57.2 ± 13.4	(54.1-60.4)	53.7 ± 21.4	(44.9-62.4)	.686
6 mo	15.2 ± 15.7	(11.5-18.9)	17.3 ± 11.7	(12.5-22.1)	.290
Last follow-up	8.7 ± 14.7	(5.2-12.1)	7.1 ± 9.3	(3.3-10.9)	.899
ASES score					
Baseline/preoperative	53.6 ± 15.6	(49.9-57.2)	52.2 ± 28.2	(40.7-63.8)	.191
6 mo	78.3 ± 14.9	(74.7-81.8)	75.6 ± 9.9	(71.6-79.7)	.117
Last follow-up	90.3 ± 14.0	(87.0-93.6)	91.5 ± 9.3	(87.7-95.3)	.927
Constant score					
Baseline/preoperative	53.7 ± 16.2	(49.9-57.5)	51.8 ± 14.8	(45.8-57.9)	.759
6 mo	75.5 ± 14.9	(72.0-79.0)	73.0 ± 8.5	(69.6-76.5)	.118
Last follow-up	83.0 ± 14.4	(79.6-86.4)	80.1 ± 9.9	(76.0-84.1)	.064
SANE score					
Baseline/preoperative	51.8 ± 16.2	(48.0-55.6)	44.3 ± 18.3	(36.9-51.8)	.077
6 mo	79.9 ± 15.7	(76.2-83.6)	78.1 ± 11.1	(73.6-82.7)	.336
Last follow-up	88.8 ± 17.5	(84.7-92.9)	92.7 ± 9.8	(88.7-96.6)	.502
Sugaya at 6 mo					
Type 1	49 (71.0%)		14 (60.9%)		.562
Type 2	12 (17.4%)		6 (26.1%)		
Type 3	5 (7.2%)		1 (4.3%)		
Type 4	1 (1.4%)		0 (0.0%)		
Type 5	2 (2.9%)		2 (8.7%)		
Satisfaction at 6 mo	59 (85.5%)		21 (91.3%)		.723
Work return at 6 mo∗					.437
No	3 (5.7%)		0 (0.0%)		
Yes	45 (84.9%)		15 (100.0%)		
Planified	5 (9.4%)		0 (0.0%)		

Bold value indicates statistical significance.

ASES, American Shoulder and Elbow Surgeons; CI, confidence interval; LHB, long head of the biceps tendon; SANE, Single Alpha-Numeric Evaluation; SD, standard deviation; VAS, visual analog scale.

∗ Retired patients were excluded for this specific analysis from the avulsion (n = 16) and Fosbury flop (n = 8) groups.

**Fig 4:**
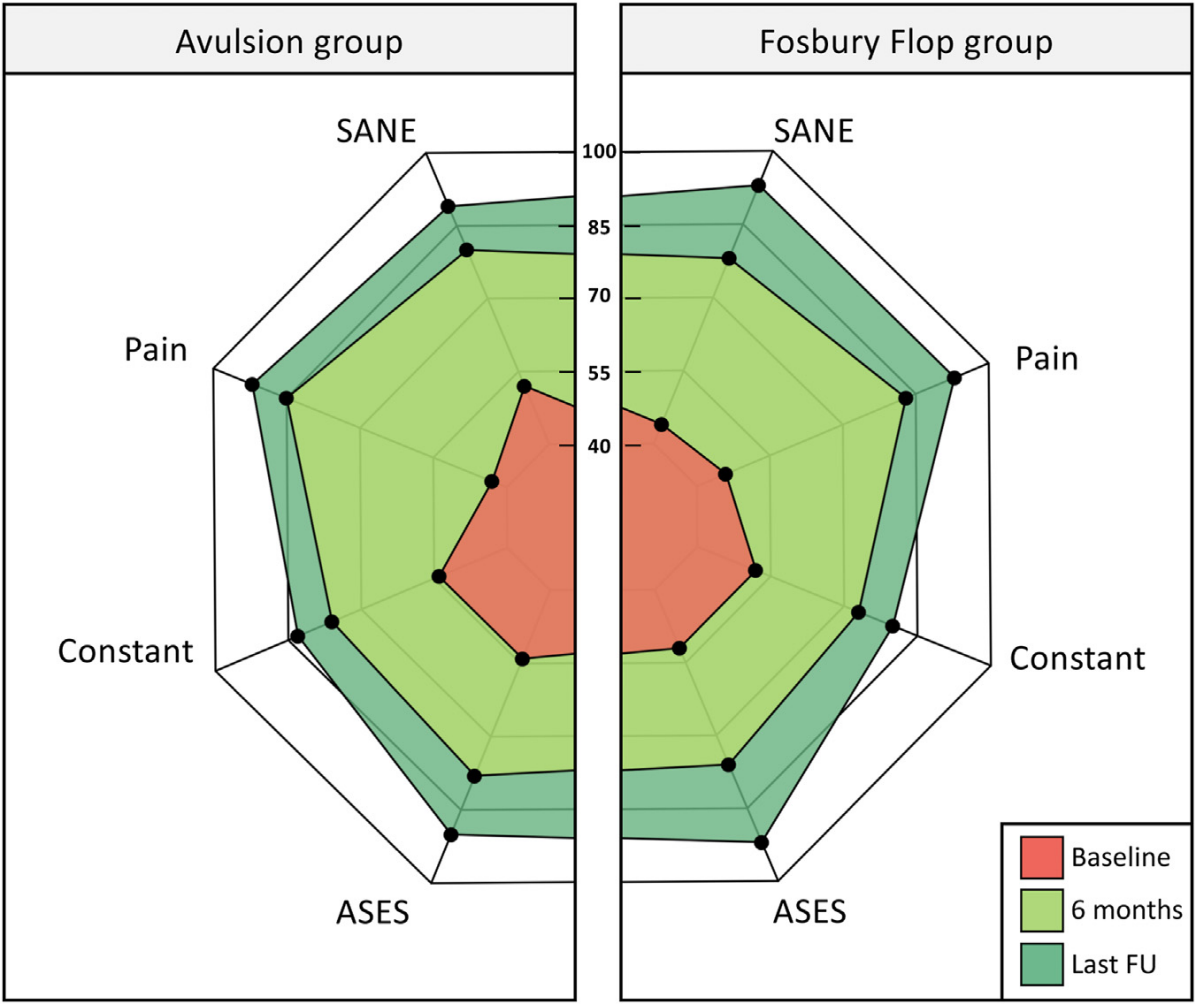
Shown are comparable clinical outcomes after arthroscopic repair of Fosbury flop tears and tendon avulsion at 6 months after the intervention and at last follow-up. (ASES, American Shoulder and Elbow Surgeons; FU, follow-up; SANE, Single Alpha-Numeric Evaluation.)

Both groups demonstrated significant improvement in ROM, VAS pain, Constant, SANE, and ASES scores at 6 months and last follow-up (Fig 4). There was no significant difference in the ROM and clinical scores between the groups at 6 months and last follow-up (Table 2). It is also worth mentioning that the Fosbury flop group did not significantly differ from the avulsion group in terms of patient proportion exceeding the ASES MCID (96% vs 94%, *P* = 1.000), substantial clinical benefit (96% vs 84%, *P* = .282), and patient acceptable symptom state (74% vs 79%, 775). *P* = .Likewise, there was a nonsignificant difference in retear rate with 8.7% (2/23) in Fosbury flop group compared with 4.3% (3/69) in avulsion group (*P* = .562).

## Discussion

This study demonstrates that the clinical and radiologic outcomes of Fosbury flop are comparable with standard avulsion of full-thickness rotator cuff tears after arthroscopic rotator cuff repair, invalidating our hypothesis. Both groups of patients reported significantly improved ROM, VAS, Constant, ASES, and SANE scores at 6 postoperative months and at last follow-up (approximately 4 years after surgery).

There was a high rate of tendon healing in the Fosbury flop tears group (91%). This indicates that the morphology of a tendon flipped onto itself does not affect healing and functional outcomes after surgical repair. As such, the key importance of understanding and recognizing a Fosbury flop tears is to ensure that an appropriate surgical dissection and reduction is performed, coupled with repair of the flipped tendon.

Everted bursal-sided partial-thickness rotator cuff tears share many morphologic similarities to the Fosbury flop tears but differ in that they are full-thickness rotator cuff tears. Everted bursal-sided partial thickness rotator cuff tears have been previously studied. Similar to the Fosbury flop tears, Kim et al.^31^ found that these tears had tendon stumps that were thickened due to folding of the tendon and retracted in a superomedial direction. Kim et al.^31^ compared everted bursal-sided partial thickness rotator cuff tears to simple rotator cuff tears and found that patients with the everted type had greater preoperative pain levels and significantly lower preoperative flexion and abduction. Everted-type tears were more difficult to approximate and more often required additional sutures compared with simple-type partial tears (64% vs 16%). When compared with simple partial-thickness rotator cuff tears, the everted type had comparable, satisfactory clinical and structural outcomes at final follow-up.

The Fosbury flop pattern and arthroscopic characteristics share many similarities with the everted bursal-sided partial rotator cuff tears and may potentially result from progression of the partial tear. Like the everted bursal-sided partial rotator cuff tears, Fosbury flop repair can be challenging because it requires meticulous dissection of the adhered flap. If the adhered flap is not well preserved, there may be increased difficulty in flipping the tendon back onto the footprint. In addition, chronicity may affect the morphology of the leading edge of the tendon tear. Akin to chronic meniscal tears, the rotator cuff tear can become deformed and bulbous over time.^32^ This leads to additional difficulty in achieving a “perfect fit” when reducing the edge of the tear to the footprint. As such, even after initial repair, the tendon edges may still present uneven tendon margins and require additional sutures for more accurate tendon approximation. One advantage of the Fosbury flop tears is that, upon adequate and meticulous release of the healed tendon flap, the torn tendon may easily be flipped back onto the humeral head footprint. Additional releases are not commonly required. As overtensioning can result in rotator cuff repair failure, this characteristic of Fosbury flop repairs may allow for cuff repair without excessive tensioning.^33^^,^^34^ Despite some technical challenges, it would appear that Fosbury flop tears, like the everted bursal-sided partial rotator cuff tears, demonstrate similar clinical results after repair when compared to tendon avulsion.

The literature also describes everted bursal-sided delaminated supraspinatus tears.^35^ Intraoperatively, they have multiple, pedunculated lesions on the bursal flap and these have been termed by Kim et al.^35^ as a “broccoli-like sign.” This is similar to the “sea-anemone” appearance^2^ and is likely of a similar pathogenesis. Kim and Jung^35^ correlated the everted bursal-sided delaminated tears to higher preoperative pain levels. Still, interestingly, the clinical outcomes of such repaired tears were significantly superior to classic delaminated tears. This further supports our findings that bursal eversion of a rotator cuff tear is unlikely to confer negative clinical outcomes after arthroscopic repair as long as the dissection plane is found.

Clinical results of Fosbury flop tears have been studied by other authors. Kajita et al.^3^ found in their study of 33 patients with Fosbury flop tears, a significantly greater retear rate of 15.2% compared with a matched cohort of 52 patients with avulsion and a retear rate of 1.9%. There are several possible explanations for this difference. First, this study does not mention the presence and grading of fatty infiltration, which has been demonstrated to affect rotator cuff retear rates.^36^ In their study, Kajita et al.^3^ matched the cohorts by tear size, yet there was no mention of the stage of fatty infiltration. This is an essential factor that must be accounted for. A second element possibly affecting the studies regarding Fosbury flop tears is a potential missed diagnosis. It is likely that surgeons may have encountered Fosbury flop tears before their published description. Nakamizo^37^ has previously described subacromial incarceration for a torn rotator cuff, which is in essence a Fosbury flop tears. In addition, whereas preoperative imaging findings have been well described on MRI,^4^ these features have not yet been defined and validated for other forms of imaging, such as ultrasound. Thus, for patients with only preoperative ultrasound scan, surgeons may not be aware of the presence of a Fosbury flop tear and overlook intraoperative signs of this lesion, such as the typical “sea-anemone” appearance. This may affect the analysis of the outcomes of such tears. The other study by Nakamizo^37^ reported 17 patients that confirmed our findings. Nakamizo^37^ observed an increased in UCLA score from 11.9 ± 2.7 points to 32.5 ± 2.1 points postoperatively (*P* = .002), with healing in all cases.^37^

The etiology of Fosbury flop tears is not well understood. One risk factor may be acromial morphology. There is growing interest in the idea that acromial morphology may be related to the presence of rotator cuff disease, although causation is yet to be clearly determined.^38^, ^39^, ^40^ With regards to Fosbury flop tears specifically, a recently published article correlated these lesions to the presence of inferior osteophytes within the central acromion (termed as a double-floor acromion in the coronal plane).^3^ Acromial morphology was also found to be associated with everted bursal-sided partial rotator cuff tears.^31^ However, it is essential to note that these acromial morphologies are largely descriptive in nature. Further studies should be performed to confirm the association between acromial morphology and Fosbury flop tears .

This study has found that the functional and clinical outcomes of arthroscopic repair of Fosbury flop tears are satisfactory and comparable with tendon avulsion at a mean follow-up of 4 years. Although the pathophysiology of Fosbury flop tears is currently unclear, the healing of the flipped fragment of the tendon onto itself may suggest some degree of vascularity of the fragment. These tears also tend to have less retraction than standard avulsion, perhaps explaining their excellent prognosis. Finally, by maintaining a high degree of suspicion for Fosbury flop tears, surgeons can effectively restore the tendon to its original footprint without excessive tension. These factors may contribute to favorable clinical outcomes after arthroscopic repair.

### Limitations

This study presents some limitations. First, as a retrospective study, some biases may exist based on the study design. Second, the sample size of Fosbury flop tears is relatively small (notably due to the inherent rarity of the pathology), although it remains sufficient for our study as underlined by the a priori sample size calculation. The strength of this study is that the surgeon was well-versed in Fosbury flop tears, likely limiting the number of missed diagnoses. In addition, Fosbury flop tears were repaired in a standardized fashion with similar postoperative protocols, allowing for a more robust assessment of the clinical outcomes after arthroscopic repair. Another limitation of this study was the assessment of preoperative characteristics of the rotator cuff tear on MRI. It has been established that there is only moderate reliability in the assessment of fatty infiltration and only moderate-to-high reliability in the assessment of tendon retraction on MRI. Although this reliability could improve by repeated assessment at different time points, this was not feasible in this study.

## Conclusions

Arthroscopic rotator cuff repair of medial bursal-side Fosbury Flop rotator cuff tears results in favorable clinical and radiologic outcomes at 4 years after surgery. These outcomes are comparable with surgically repaired avulsion lesions, with an acceptable retear rate after arthroscopic repair.

## Disclosure

The authors declare the following financial interests/personal relationships which may be considered as potential competing interests: FORE (Foundation for Research and Teaching in Orthopedics, Sports Medicine, Trauma, and Imaging in the Musculoskeletal System) grant # FORE 2023-52. A.L. is a paid consultant for 10.13039/100008894Stryker, 10.13039/100007307Arthrex, Medacta, and Enovis. He received royalties from Stryker. He is the founder of FORE, BeeMed, and Med4Cast. He owns stock options for Medacta and FollowHealth. He is on the board of the French Arthroscopic Society. P.C. has received personal fees from Enovis, and Stryker. He is cofounder of Med4Cast and Follow. He is on the board of SECEC and IBSES. All other authors (S.W.L.H., T.M., A.A., J.Z., X.L.C., H.B., P.C.) report no conflicts of interest in the authorship and publication of this article. Full ICMJE author disclosure forms are available for this article online, as supplementary material.

## Supplementary Data

ICMJE author disclosure forms
